# Happy 25th birthday, Bet v 1!

**DOI:** 10.1186/1939-4551-7-14

**Published:** 2014-06-03

**Authors:** Erika Jensen-Jarolim

**Affiliations:** 1 Comparative Medicine, Messerli Research Institute, University of Veterinary Medicine Vienna, Medical University Vienna and University Vienna, Waehringer G. 18-20, 1090 Vienna, Austria; 2 Institute of Pathophysiology and Allergy Research, Waehringer G. 18-20, 1090 Vienna, Austria

## 

As the deputy editor of WAOJ I am concentrating on basic science, but with continuous focus on the clinical relevance of submitted studies for our allergic and atopic patients. The questions that follow me are 1.) “What makes an allergen an allergen?” and 2.) “Why do humans and other mammalians form IgE?”.

Within the period which I personally can oversee in the allergy field, i.e. roughly 25 years, tremendous efforts and luckily also progress have been made in understanding allergy mechanisms, from the Th1-Th2 [1] paradigm to Tregs [2], from immunoblot [3] and molecular cloning of allergens [4-6] to cloning of IgE receptors [7,8], the cognition of their function in antigen presentation [9,10] and transgenic models imitating human disease [11]. This time span covered the transition from allergen extracts to molecular allergology. The latter is perhaps the most significant change that we experienced, which so far at least resulted in the improved diagnosis of allergies [12]. We may today decide between molecules causing less or more severe symptoms and between allergens that are associated with the different clinical pictures. This means true help for optimal patient care.

For instance, Fel d 1 from cat and Can f 1 from dog are significantly associated with asthma and may be regarded as biomarkers for it [13]. Not only molecules, but also carbohydrate decorations like alpha-Gal may cause severe symptoms – and be associated not only with pet and insect sensitization, but also with severe food allergy, or –amazingly, with hypersensitivity reactions to biological agents when expressed in insect vectors [14].

From the first analyses of allergen extract we have started to experience that not all, but a limited number of compounds act as allergens, i.e. form and bind IgE [15]. Why is that so? At least for some molecules research has so far brought out the interesting information that sensitization may have to do with the function of the molecules.

For instance, Der p 1 from house dust mite, which enzymatically breaks up the bronchial and skin barrier, thereby promotes its own entrance [16]. Hence we understand why for some allergens the risk for sensitization is higher the higher the exposure, even better in atopic predisposition. This approves our empiric observation that allergen reduction in the environment, or better avoidance, is an important practical measure. In fact, we thereby also control their non-specific action. Some allergens are inhibitors of enzymes, and one might speculate that they inhibit the gastrointestinal digestion and may persist during the transit due to their own inhibitory function. For instance, Ara h 2 is a trypsin inhibitor and food processing such as roasting may even enhance these functions [17].

One of the most chased allergen, however, is Bet v 1. Here, the situation is even more extreme: Bet v 1 is the single major allergen from birch pollen. I am proud to say that Vienna is the cradle of one of the most important and fascinating molecules in allergy: Right from the beginning Bet v 1 was classified as a pathogenesis-related molecule being overexpressed in various stress conditions of the plant [4]. Since its cloning in 1989, its T-cell and B-cell epitopes were defined [18,19] and the Bet v 1 structure was revealed by X-ray crystallography as a 17 kDa globular molecule with a pocket inside [20]. We understand that the birch pollen related oral allergy syndrome is due to molecular homology causing IgE-crossreactivity among the Bet v 1 homologs in many plant species which we eat [21]; the fact that Bet v 1 and most of its food homologs are devoid of intramolecular disulfide bridges explains their lability in the gastrointestinal transit [22] and makes them “non-sensitizing elicitors” of food allergy. Whether multiple epitopes are needed for crosslinking in the effector phase or whether dimer formation of Bet v 1 is needed for its IgE cross linking capacity, is a matter of debate [23,24]. In search for the ligand of the secret intramolecular pocket of Bet v 1, researchers recently proposed quercetin-3-O-sophoroside, to make the fit [25]. However, the basal question remains: why is exclusively Bet v 1 the allergen out of the plethora of birch molecules in the extract? Why is Bet v 1 a unique inducer of primary sensitization, of a significant Th2 bias and isotype switch to specific IgE production?

**Figure 1:**
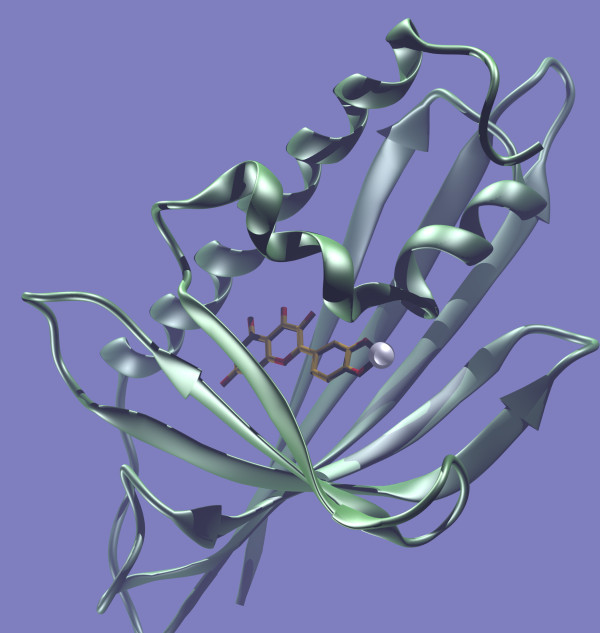
**The Betv1-Fe-quercetin complex.** The Birthday-molecule Bet v 1 (silver), carrying a siderophore molecule (quercetin) in its pocket binding one iron molecule Fe^3+^ (white ball). Bet v 1 acts as allergen when its pocket is devoid of iron. Cartoon kindly created by Prof. Luis F. Pacios, Unidad de Química y Bioquímica, Departamento de Biotecnología, E.T.S.I. Montes, UPM, 28040 Madrid, Spain.

We have the answer today. Quercetin is a siderophore and as such is able to bind iron similarly to catechols. More importantly, the siderophore-iron complex is able to bind with an outstanding binding affinity to lipocalin proteins [26]. By a revolutionary approach, Bet v 1 could in fact be classified as a lipocalin-like protein [27] (Figure 1). Most importantly, Bet v 1 in the absence of Fe^3+^ manipulates Th-cells and skews the immune response towards Th2 whereas the presence of iron abolishes a Th2 response. This molecular and functional understanding contributes another stone in the allergy puzzle. Happy 25^th^ birthday, Bet v 1!

So, why is it important to bring more of molecular allergy into WAOJ? Using this journal as a global information exchange platform, we may sense system errors in our changing world, in our environment or nutrition, which are able to directly affect the immune balance. In fact, we should in a concerted action systematically search for system errors in our societies, which contribute to allergy and atopy in humans and their pets; errors that have been introduced by establishment or fall down of borders, by introduction of new technologies in traffic or food production. WAOJ should be a platform specialized for collecting data from all over the world elucidating geographical differences in molecular sensitization patterns between countries, south and north, or even between neighboring villages.

We are looking forward to receive your submissions.

Warmest,

Erika Jensen-Jarolim
